# Optimizing the cost of the STEP programme

**DOI:** 10.1098/rsta.2023.0413

**Published:** 2024-10-09

**Authors:** Hanni Lux, Chris Brown, Matthew Butcher, Rhian Chapman, Jack Foster, Nousheen Nawal

**Affiliations:** ^1^ UKAEA (United Kingdom Atomic Energy Authority), Culham Campus, Abingdon, Oxfordshire OX14 3DB, UK

**Keywords:** costs, cost optimization, fusion power plants, STEP

## Abstract

The Spherical Tokamak for Energy Production (STEP) programme is a world-leading fusion power plant programme that has embedded a cost conscience in its design from the early phases. This firmly addresses the attitude of cost complacency of which many major infrastructure projects have historically been accused. While a detailed and highly accurate whole life cycle cost analysis is not possible, or even valuable, during the conceptual design stage, this early design phase is still the most critical programme phase where a focus on costs can drive longer term reductions and impact whole life cycle costs at the high level. Consequently, appropriate estimating methods for these early-stage designs and lessons learned from other industries are used to inform design decisions and ensure cost is part of the overall option analysis. Hence, while the overall programme cost estimate is too immature to be a reliable indicator for the final programme costs, significant effort has been undertaken to understand the major cost drivers and take action to make the STEP design as cost-effective as possible.

This article is part of the theme issue ‘Delivering Fusion Energy – The Spherical Tokamak for Energy Production (STEP)’.

## 1. Introduction

Whole life as well as capital cost analyses of fusion power plant concepts have been published in the literature since the early days of fusion power plant conceptual designs (e.g. [1–7]). However, not all conceptual fusion power plant designs are accompanied by corresponding cost estimates (e.g. [8]) or have only published partial cost models focused on individual reactor components’ capital costs (e.g. [9]). Given the major uncertainties involved, both in design detail and corresponding cost estimates, this is unsurprising. Nevertheless, the early stages of design offer the greatest opportunity to embed cost consciousness and understand the factors that will, in time, lead to more cost-effective solutions. The Spherical Tokamak for Energy Production (STEP) programme has been systematically designed with cost in mind at every stage of the process [10], making STEP unusual, if not unique, in the endeavour to optimize fusion power plant design for future costs. A holistically applied cost conscience is considered critical to fulfilling the STEP mission to ‘deliver a UK prototype fusion energy plant, targeting 2040, and a path to the commercial viability of fusion’.

While the maturity of whole-life cost assessments in early design phases is expected to be low, taking cost into account during design decisions at that phase of the design process is critical as it offers the greatest opportunity to have a positive impact on the cost of the programme in the longer term (e.g. [11]). This is even more important for fusion, which is a novel, low-carbon technology aiming to penetrate the international energy market, but which is often perceived to be non-commercially optimized for electricity generation (e.g. [12]). Furthermore, sufficiently low capital costs are expected to be a crucial factor in the predicted market penetration of fusion [13].

One of the main drivers of fission power’s move towards small modular reactors [14] is the recognition that typically large mega-projects have significant cost and schedule overruns unless they have been designed in a modular fashion [15]. The STEP programme is, therefore, consciously considering different types of modularity and standardization both in its design and subsequent build of the STEP prototype plant (SPP) and is actively probing the smallest plant size that is capable of electricity production and a path to commercial viability. In that context, the STEP programme notes that while fusion has traditionally targeted the electricity market commercial viability for fusion might lie in other uses [16]. As a result, the programme aims to keep development flexibility to be able to provide other useful outputs.

This work first illustrates how STEP takes best practice estimating methodologies into account when creating cost estimates for the entire SPP and how it has adapted these for STEP-specific requirements. It then shows how the cost estimates have influenced the design to an optimal point within the wider trade-off space, given the limitations of the large uncertainties present at this stage. Finally, it discusses challenges with the current analysis and gives an outlook on future work.

## 2. Methodology

The STEP cost team holistically assesses all costs expected to be incurred by the STEP programme from programme stage Tranche 2 a (first part of the STEP engineering design phase starting in April 2025) onwards, including the detailed engineering design programme and construction of the SPP, as well as the wider life cycle costs of operations and decommissioning beyond the end of the STEP programme.^1^ However, during the conceptual design phase supporting choices with differential cost assessments (together with creating more detailed cost models for the next funding tranche) is the most critical aspect of the costing work. The high-level costing methodology used for differential cost assessments is drawn from best practices across multiple industries and has initially been described in [16]. Changes to the methodology since the publication of [16] cover the modification of the cost breakdown structure (CBS) that has been changed to adapt to the programme-specific needs as the programme shifts towards using the CBS for cost control and management from Tranche 2a onwards.

Estimating methods for the individual subcomponents of the CBS covers a range of techniques appropriate to the maturity of the design: top-down, estimation by comparator, parametric scaling, relevant subject matter expert knowledge/judgement, etc. (see table 1 for typical early-phase estimation methods). Each part of the cost model is reviewed as the design matures and estimating methods are adapted with respect to the maturity of the design. The specific tools used to transition from predominantly top-down to more bottom-up cost modelling are described in more detail in [18]. The cost team currently uses both the PROCESS systems code [19,20], which allows fast assessments of cost impacts on a variety of design options and the commercially available CALC4XL tool. CALC4XL is an add-on to Excel that enables combining different estimating methods for each sub-part of the CBS, down to full bottom-up cost modelling from standard and bespoke manufacturing steps, detailed CAD drawings for material volumes, scrappage, overheads, etc.

**Table 1.** Association for the Advancement of Cost Engineering (AACE) cost estimate classes [17] showing a selection of typical estimating methods used for different levels of project definition as well as corresponding accuracy ranges. Capacity-factored estimates calculate the cost of a new project based on a scaled cost of an existing project with a different capacity. Equipment-factored estimates multiply the cost of the known equipment by a factor taking installation costs into account. Detailed unit costs with detailed take-off are cost estimates using detailed drawings, where the cost estimator assures completeness and no double counting of all materials and labour cost components. It requires a complete bill of quantities.

estimate class	low	high	methodology
**class 5**	−50%	+100%	capacity factored, parametric models, judgement or analogy
**class 4**	−30%	+50%	equipment factored or parametric models
**class 3**	−20%	+30%	semi-detailed unit costs with assembly level line items
**class 2**	−15%	+20%	detailed unit costs with forced detailed take-off
**class 1**	−10%	+15%	detailed unit costs with detailed take-off

Table 1 also illustrates the overarching changes in the expected accuracy range of the cost estimates as the programme matures. While the overall trend of the accuracy of the STEP programme cost model is expected to mature through the typically used estimating classes [17], there are areas of the cost model (such as where the design is similar to existing power plants and, therefore, more mature) where the cost accuracy can be expected to be better than for the more fusion-specific costs. However, owing to the innovative nature of significant parts of any fusion power plant design, fusion island components, i.e. the components relevant for the fusion-specific heat production, costs are expected to be significantly less accurate than those of other engineering design programmes at the same stage of the design. The cost modelling uncertainties are expected to decrease as various parts of the STEP programme mature; this is described in [21].

In addition to uncertainties, the impact of identified risks on costs, both the cost of any mitigation activities and the impact of unmitigated or residual risks occurring, will be included in the overall cost model, once it is sufficiently mature. Furthermore, it is critical to consider an optimism bias correction when assessing the whole programme cost in the early stages of such novel technology programmes. This is addressed using reference class forecasting and the alignment of our approach with best practices as described in [22].

While it is not appropriate to estimate the levelized cost of electricity (LCOE) for a prototype plant, which will not be operating in a commercially relevant regime, operational costs do need to be assessed for extrapolation to commercial power plants, to understand the cost drivers for those future programmes and to evaluate the legacy costs of the SPP. In this context, it is important to note that a prototype plant has many cost items that are not shared with Nth-of-a-kind (NOAK) commercial plants, i.e. the engineering design programme is significantly more extensive, additional tests and prototypes are required, but also the costs of developing a fusion relevant supply chain need to be covered by the STEP programme, unless synergies with other fusion programmes can be found. Furthermore, costs of many fusion-relevant items are expected to reduce for NOAK commercial plants owing to learning by doing cost reductions in an industrialized supply chain [23].

To make well-balanced cost decisions, the STEP programme aims to address costs as best as is feasible, in a holistic and integrated fashion [24], while considering the impact on NOAK commercial plants [25]. This supports the aim of the STEP programme to have a clear pathway to commercialization.

## 3. Cost conscience in the programme

For all the reasons previously mentioned, it is essential for a programme like STEP to be set up with a strong cost culture. The way this culture is reflected in the programme has been demonstrated from the early concept stages:

— STEP has been consciously chosen to be a spherical tokamak design under the assumption that this will help reduce the reactor size and therefore costs. This is a strategic decision that has been taken in the early stages of STEP to focus the programme. Owing to the current lack of maturity of whole programme cost estimates for any fusion power plant, more work needs to be carried out to validate this assumption.
— The STEP mission is to ‘deliver a UK prototype fusion energy plant, targeting 2040, and a path to the commercial viability of fusion’, where the latter part requires a cost conscience during design.
— The programme measures of effectiveness (MoE) [10] are constraining among other aspects both the capital (CAPEX) and operational (OPEX) cost for the SPP.
— Both CAPEX and OPEX requirements have been captured for the plant and will be constrained to absolute values once the cost estimates are sufficiently mature. These will be propagated down to individual control accounts for each major component.
— During a major design review of the STEP design held in autumn 2021, the STEP director formulated multiple principles of design, two of which are [26]:
  - Small as possible (lowest cost) while delivering MoEs, balanced by: resilience—margin to achieve confidence.^2^
  - Simplicity: drives reliability and cost.
— Decision capping reports, i.e. internal reports that record the reasons why a design decision has been taken as it was, are requested to either include a cost evaluation section—where expected to be impactful—or to motivate why costs were not considered to be a distinguishing factor.

The combination of the above points has initiated, from the highest levels of the programme, a directive to address cost as part of the whole design process.

## 4. Addressing major cost drivers

While the total programme costs of the STEP programme are still immature, most decisions influencing the cost of the design have been taken on differential or qualitative cost assessments, which are more reliable at this stage of the programme. As a result, comparisons with other fusion programmes or radically different design choices cannot be addressed, but the design itself is ensured to be as cost optimized as it can possibly be within the existing uncertainties.

The cost model team has focused their efforts on the high-impact areas, i.e. those of expected high costs, of high cost and high uncertainty, and areas where clear options are available. Therefore, the list below is not exclusive but aims to highlight the impact of the most significant factors on costs.

### (a) Programme scope

Defining the programme scope has the biggest impact on overall programme costs. The scope of the STEP programme includes the design and creation of the STEP prototype power plant, the relevant technology demonstration and research to underpin the design, the development of the corresponding supply chain and any activities necessary to ensure a pathway to the commercial viability of fusion, not already demonstrated on the SPP. All of these aspects are essential and cannot be excluded for the STEP mission to be successful. While STEP is consciously creating a company limited by shares (UKIFS Ltd), to allow multiple funding sources, it has specifically chosen a structure that does not allow funding through contributions in kind as in the International Fusion Experiment in the South of France (ITER) model, which is optimized for the democratization of growth and intellectual property instead of cost efficiency. As the STEP programme matures, opportunities for synergies with other programmes might be realized that can significantly reduce the overall cost of the programme without reducing its benefits. As a result, the STEP programme considers its current scope and operating model as cost optimized as it can be for such a complex first-of-a-kind (FOAK) endeavour.

### (b) Regulatory approach

While the UK regulatory approach is not determined by the STEP programme, it nevertheless has a significant impact on the costs of the SPP. Anecdotal evidence from ITER suggests a significant increase in the costs owing to the requirement to follow the nuclear fission regulation, which has a much higher intrinsic safety hazard. Though the magnitude of the cost impact cannot be quantified, the STEP programme will benefit from the proportionate regulatory approach taken in the UK [27,28] as it ensures the cost efficiency of the industry without jeopardizing safety. (In the UK, fusion energy will not be regulated by the Office for Nuclear Regulation and its intrinsic and more passively safe nature is taken into account.) As addressed in [16], global harmonization of fusion regulation is the next step towards a globally competitive fusion energy production. However, this is outside the delivery scope of the STEP programme.

### (c) Engineering approach

The relation of the impact of investment in systems engineering on cost overruns for NASA has been well documented [29]. Furthermore, poor information management has been shown to be one of the risk factors leading to optimism bias in the procurement of equipment/development [30]. These are just two examples where the STEP programme uses lessons learned and best practices from other programmes to control costs [31].

### (d) Tokamak size

**Figure 1.**
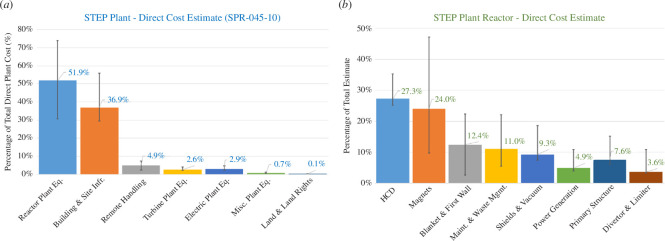
Illustration of the relative direct cost including uncertainties identified in the CML5 design point (SPP-045-10) for both the breakdown in total plant direct costs (*a*) and reactor equipment direct costs (*b*). The data is available from https://doi.org/10.24447/b24f-n554.

To highlight the importance of design size on overall programme costs, figure 1 illustrates the main cost components in the direct costs, i.e. costs associated on an item-by-item basis with the equipment and structures that comprise the complete power plant including permanent service and support facilities, for both the total plant and the reactor equipment. As can be seen, the overall direct costs are dominated by the reactor equipment and building costs. The building costs are dominated by the reactor building and tokamak logistics centre costs, which both scale directly with the size of the tokamak; additionally, the reactor equipment costs are made up in a significant portion by components that scale with the size of the tokamak, such as the magnets, blanket and first wall, vacuum vessel and primary support structure, etc.

As already highlighted in §3, the programme aims to minimize the plant design size within margins for resilience, while fulfilling the project goals explicitly to constrain programme costs. There is no good reason for the SPP to be any larger, and therefore more expensive, than is required to demonstrate energy production and a path to commercialization. STEP is therefore consciously probing the smallest reactor size possible without increasing costs by increasing complexity through overcrowded spaces. A significant effort has been made to (i) understand the size drivers of the SPP, which has been found to be driven by the minimum viable inboard radial build and (ii) to probe what the minimum inboard radial build is [10].

### (e) Plasma performance

The projected plasma performance also significantly drives the tokamak size, and therefore cost, for a given fusion power/net electric output. The STEP design has consciously chosen the most efficient steady-state plasma mode [32], maximally controllable elongation [33] and appropriate impurity seeding, owing to their significant impact on costs [34]. Furthermore, choosing to operate in high normalized beta/Troyon factor β_N_ above the no-wall limit (with resistive wall mode control) and at high plasma triangularity optimizes the pedestal performance and therefore cost per fusion power [35]. This assures that the plasma performance as a significant size and cost driver is as optimized as it can be within the remaining significant uncertainties. The authors note that tokamak plasmas require active control. This is not unusual for power plants and other complex technology systems such as processing plants, the electric grid, etc. In particular, active control of the resistive wall mode (RWM), as required to operate above the no-wall limit, is equivalent to the control of vertical displacement events. Robust RWM control has been demonstrated in tokamaks able to operate above the no-wall limit. A burning plasma poses a significant control challenge as it is a highly nonlinear, strongly coupled system. The RWM control is only one aspect of the control requirements.

A cost, benefit and risk analysis of RWM control, if technically realizable, is favourable. The alternative would be to increase the size of the device, thereby substantially increasing the plasma risk, to increase the plasma elongation making vertical control more challenging or incompatible with a tritium breeding ratio (TBR) > 1.1 or to increase the toroidal field strength beyond what is achievable within the space constraints of the centre column.

### (f) Heating and current drive

Heating and current drive is currently forecast to be one of the more expensive reactor systems. Furthermore, it is the highest source of parasitic power load for the steady-state STEP plant. Every effort has been made to choose the highest efficiency to drive current during the plasma’s steady state regime (i.e. the product of the grid to plasma electrical efficiency and the current drive efficiency), which has the biggest impact on the overall design cost and the best net electric output [34]. The current choice of HCD technology is electron cyclotron resonance heating with electron Bernstein waves as an even higher efficiency opportunity [35], should it be shown to work in a burning plasma environment. Furthermore, significant cost reduction and efficiency enhancements are expected during the next 10 years prior to the start of the build [36].

### (g) Re-mountable magnets and maintenance strategy

Re-mountable magnets are considered a clear enabler of a commercially relevant assembly and maintenance regime that is required to achieve competitive LCOE. Additionally, they are one way of reducing the required piece length of the conductor tape, thereby reducing wastage and in turn manufacturing costs [37].

### (h) TF coils conductor choice

Contrary to initial expectations, a more holistic assessment of the toroidal field (TF) coil system costs (magnets and required cryo-plant) has shown that within the current uncertainties, costs are not a differentiating factor between the cryogenic aluminium and high temperature superconductor (HTS) TF coils [24]. (Differential cost data for power supplies between the two different choices were not available at the time of the decision and have therefore not been considered. Subject matter experts suggest that the costs are smaller than the conductor and cryo-plant costs and are unlikely to skew the results significantly.)

### (i) Blankets

As the blankets are significant cost components of the reactor, the materials-based cost model for the blanket has been built into the design optimization calculations, ensuring that cost is always considered. The blanket cost model currently assumes a helium-cooled blanket with a liquid lithium breeder layer. In a non-liquid lithium breeder configuration, previously, Eurofer 97 was the major cost driver of the component as a structural material, with the switch to a vanadium alloy and a liquid lithium breeder layer, the metallic lithium breeding zone has been the biggest cost driver in the blanket. The authors note that, owing to the non-existent industrial supply chains for either Eurofer 97 or relevant vanadium alloys, the cost uncertainties in both materials are larger than the expected cost differences between them. While a similar or even worse challenge applies to the costs of enriched liquid lithium, we find that the expectation that enriched liquid lithium costs will dominate over the structural material costs is likely to hold true. This is owing to the undeveloped market in liquid lithium and the undetermined and likely complex process of 6Li enrichment. The highly reactive nature of liquid lithium also adds significantly to the handling and installation costs.

While total life cycle costs would be the correct comparator when making cost decisions, owing to the lack of definition of the operating regime and the resultant significant uncertainties in operational costs, often capital costs are used as a decision driver instead in an attempt to assess the cost impact in a more holistic fashion. Though the material cost of the vanadium alloy is expected to be significant, it is expected to have a longer lifetime than alternative alloys, decreasing the replacement frequency and in turn the operating costs. This leads to a lower life cycle cost and LCOE. Furthermore, the use of a vanadium alloy as the outer first wall and breeder blanket structural material has a positive impact on the tritium breeding ratio owing to better neutron characteristics.

### (j) Divertor performance

While the divertor is not a significant size driver for the SPP design (which is driven by the inboard radial build), it is expected that the divertor performance might be the critical size and therefore a cost driver for the commercial plant design [25,38], hence divertor performance has been optimized by choosing a double-null, long-legged divertor with a liquid tin surface [39].

### (k) Maintenance strategy: repair versus replace

A cost-benefit analysis was performed to determine the right balance between repairing or replacing a component in the event of failure with certain repair scenarios recommended for further technical investigation, as they carry significant cost-saving benefits. Initial results suggest that repairing components with unexpected failures is probably only cost beneficial before low-activation commissioning starts, after which replacing components is expected to be more cost efficient. It is expected to be more beneficial to replace in-vessel components with end-of-life failures at any point.

## 5. Discussion and key challenges

Lessons learned in the MIT study [40] state that ‘early-stage cost estimates are unreliable predictors of the eventual cost of mega-projects’. Furthermore, lessons learned from the NCSX programme [41], which was a proposed, medium-sized, compact stellarator plasma physics experiment, suggest that baselining cost estimates for fusion projects prior to significant prototyping of bespoke fusion components can lead to unpredictably large uncertainties in cost estimates. STEP is a mega-project and a FOAK fusion device, it is therefore sensible to delay the initial baselining of the total programme costs until the later phases of Tranche 2a.

Often the cost of fission power plants is used to compare against the cost of fusion power plants, owing to their expected similar niche in the energy market and therefore to assess the commercial viability of fusion. While absolute cost comparisons with other industries are not yet possible, the FOAK nature of commercial fusion power plants can be a potential disadvantage over the mature fission technology with a mature supply chain. As a result, it is essential not to be cost negligent when designing fusion power plants. This work has illustrated multiple examples where the STEP programme has optimized costs within the current design uncertainties.

## 6. Outlook

While the STEP programme is not yet mature enough to give absolute programme costs, as the programme matures, the estimating uncertainty is expected to reduce [17]. Owing to the significant impact on total programme costs, the STEP programme has carefully embedded a cost consciousness in the programme during the conceptual phase. Therefore, it will naturally continue to not only update and mature its cost estimates but plans to continue considering the cost impact of design choices at every stage of the programme. Near-term plans of this nature include continuing to probe the space around the smallest design point that fulfils the programme requirements, has sufficient margins and minimizes costs. Furthermore, it has aimed to park design aspects that possibly add more complexity than required.

Upcoming aspects that will be critical for the design include continuing to drive modularization/standardization and the repeatability of components in the design. Using our information management system will be a critical enabler in this work [31].

## Data Availability

All data presented in this work are captured in UKAEA’s open data register and are accessible online [42].

## Footnotes

^1^
Please note that the STEP programme ends with the successful demonstration of electricity production. Therefore, the main operational/decommissioning costs are beyond the scope of STEP and are only estimated to assess the expected programme legacy costs and to support holistic cost optimization on the path to commercialization.

^2^
The current cost analysis has been carried out with holistic, but simple cost models as part of a systems code analysis. It suggests that size is the biggest cost driver of the plant. However, owing to the low fidelity nature of the analysis, it is critical to not overinterpret these results and remember that this relationship does break down, if too strong space constraints lead to the requirement for highly complex engineering solutions. Within the current uncertainties of the analysis, it is impossible to define where the turnover point will sit. Hence, it is critical to keep sufficient margins in the design to achieve confidence through resilience. This is a typical common sense adjustment required when interpreting systems code analysis.
